# Microbial Transformation of Bioactive Compounds and Production of *ortho-*Dihydroxyisoflavones and Glycitein from Natural Fermented Soybean Paste

**DOI:** 10.3390/biom4041093

**Published:** 2014-12-12

**Authors:** Changhyun Roh

**Affiliations:** Division of Biotechnology, Advanced Radiation Technology Institute (ARTI), Korea Atomic Energy Research Institute (KAERI), 1266, Sinjeong-dong, Jeongeup, Jeonbuk 580-185, Korea; E-Mail: chroh@kaeri.re.kr; Tel.: +82-63-570-3133; Fax: +82-63-570-3139

**Keywords:** screening, biotransformation, soybean paste, bioactive compound

## Abstract

Recently, there has been a great deal of remarkable interest in finding bioactive compounds from nutritional foods to replace synthetic compounds. In particular, *ortho*-dihydroxyisoflavones and glycitein are of growing scientific interest owing to their attractive biological properties. In this study, 7,8-*ortho*-dihydroxyisoflavone, 6,7-*ortho*-dihydroxyisoflavone, 3',4'-*ortho*-dihydroxyisoflavone and 7,4'-dihydroxy-6-methoxyisoflavone were characterized using microorganism screened from soybean Doenjang. Three *ortho*-dihydroxyisoflavones and glycitein were structurally elucidated by ^1^H-NMR and GC-MS analysis. Furthermore, bacterial strains from soybean Doenjang with the capacity of biotransformation were screened. The bacterial strain, identified as *Bacillus subtilis* Roh-1, was shown to convert daidzein into *ortho*-dihydroxyisoflavones and glycitein. Thus, this study has, for the first time, demonstrated that a bacterial strain had a substrate specificity for multiple modifications of the bioactive compounds.

## 1. Introduction

Doenjang is a unique traditional Korean soybean food, which is fermented by diverse microorganisms, including fungi and bacilli, during its manufacturing [1]. Doenjang made from soybeans fermentation has been studied [2,3]. In addition, three types of isoflavones and their derivatives, such as glycoconjugates and *O*-methylated isoflavones, in Doenjang were also intensely studied [4]. Daidzein, genistein, and glycitein are the most abundant isoflavone aglycones found in soy extract. Daidzein and genistein are diphenolic phytoestrogen compounds found in numerous plants, including soybeans. They have been reported to act as antioxidants, antimicrobials, free radical scavengers, metal chelators, and antibacterial agents [5,6,7,8,9,10,11].

Doenjang is known to be effective at preventing cancer, heart disease and brain tumors, and lowering blood pressure. Doenjang is rich in isoflavones and beneficial vitamins, minerals, and hormones that are reported to possess anti-carcinogenic properties [4,12,13].

**Figure 1:**
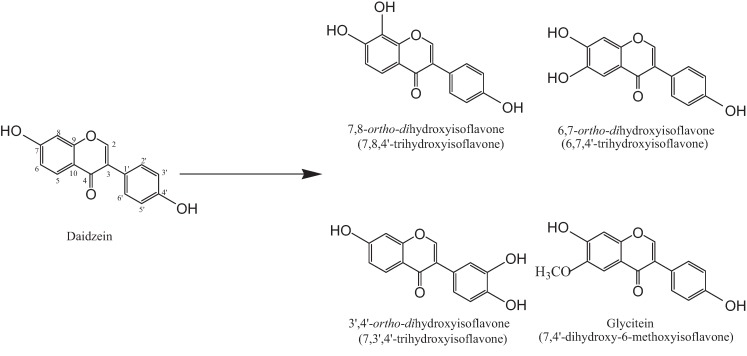
Chemical structures for biotransformation of isoflavones.

Recently, *ortho*-dihydroxyisoflavones and glycitein (Figure 1) has drawn increasing scientific interest, owing to their medicinal, chemo-preventive and nutritional properties [14,15,16]. Fujita *et al.* reported that 7,8-*ortho*-dihydroxyisoflavone is a bioactive compound with attractive pharmacological properties for the treatment of diabetic complications, such as an aldose reductase inhibitor [17]. Chen *et al.* reported that 7,8-*ortho*-dihydroxyisoflavone and glycitein have anti-mutagenic activity [18]. Rufer *et al.* described that similar compounds, 7,8-*ortho*-dihydroxyisoflavone (7,8,4'-trihydroxyisoflavone), 6,7-*ortho*-dihydroxyisoflavone (6,7,4'-trihydroxyisoflavone), 3',4'-*ortho*-dihydroxyisoflavone (7,3',4'-trihydroxyisoflavone) and glycitein (7,4'-dihydroxy-6-methoxyisoflavone), exhibited effective antioxidant activity by using an oxygen radical absorbance capacity (ORAC) assay, as well as the oxidation of low-density lipoproteins (LDL) [16].

Until now, however, there are no reports on the formation of *ortho*-dihydroxyisoflavones and glycitein, which are related to the long-time aging of fermented soybean paste. It was observed that high contents of *ortho*-dihydroxyisoflavones were specifically found in five-year-old soybean Doenjang, which led us to investigate what kinds of bacteria are responsible for such biotransformation, and whether or not the screened bacteria can do the same biotransformation in a liquid culture broth. In this study, such issues were addressed and examined for the possibility of biotransformation of such bioactive compounds using bacterial strain screened.

## 2. Results and Discussion

### 2.1. Soybean Doenjang and Amounts of ortho-Dihydroxyisoflavones Due to Fermentation Period

**Figure 2:**
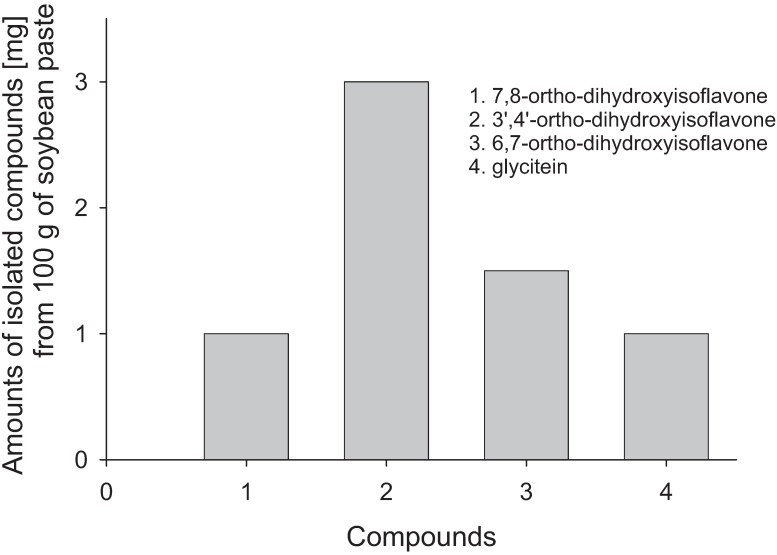
Amounts of isolated compounds from fermented soybean paste.

Korean Doenjang (Soybean paste) is made from yellow soybeans, rice, barley, and wheat. Soybeans are boiled and ground by rock into fine bits and formed into a block, called Meju, which means lumps of fermented soybeans. The blocks are then exposed to sunlight to be dried, during which mold special to soybean appears. After the blocks have been dried, they are put in a warmer place to speed up the fermentation. Next, they are put into large opaque pottery jar with brine and left for further fermentation, during which time various bacteria transform the mixture into compounds such as *ortho*-dihydroxyisoflavones. Doenjang was fermented for the biotransformation of bioactive compounds. As shown in Figure 2, *ortho*-dihydroxyisoflavones and glycitein were isolated from fermented soybean Doenjang. The amounts of purified 6,7-ortho-dihydroxyisoflavone, 7,8-*ortho*-dihydroxyisoflavone, 3',4'-*ortho*-dihydroxyisoflavone and glycitein were 1 mg, 3 mg, 1.5 mg, and 1 mg, respectively.

### 2.2. Isolation and Analysis of Isoflavone Using Microorganism Screened from Soybean Doenjang

The isolated extracts obtained from Prep-HPLC were lyophilized. For the structure analysis of the extracts, each Prep-HPLC peaks was isolated and analyzed by GC-MS after trimethylsilylation with BSTFA and ^1^H-NMR spectroscopy. The chromatographic data of the retention time, and the MS data, and the substituted functional groups of the backbone are summarized in Table 1. The mass spectra demonstrate the molecular weight of the extracts and established the distribution of hydroxyl groups for TMS derivatives between the daidzein A and B rings. ^1^H-NMR spectral data of 6,7-*ortho*-dihydroxyisoflavone: δ 8.08 (s, H-2), δ 6.91 (s, H-8), δ 7.45 (d,d, *J =* 8.7 Hz, H-2'/6'), δ 6.86 (d,d, *J =* 8.7 Hz, H-3'/5'). ^1^H NMR spectral data of 7,8-*ortho*-dihydroxyisoflavone: δ 8.32 (s, H-2), δ 7.46 (d, 8.7 Hz, H-5), δ 7.39 (d, 8.5 Hz, H-6), δ 7.45 (d,d, *J =* 8.7 Hz, H-2'/6'), δ 6.86 (d,d, *J =* 8.7 Hz, H-3'/5'). ^1^H-NMR spectral data of 3',4'-*ortho*-dihydroxyisoflavone: δ 8.37 (s, H-2), δ 7.97 (d, *J =* 8.5 Hz, H-5), δ 7.56 (d,d, *J =* 8.0 and 2.5 Hz, H-6'), δ7.53 (d, *J =* 2.5 Hz, H-2'), δ 7.42 (d, *J =* 8.0 Hz, H-5'), δ 6.95 (d,d, *J =* 8.5 and 2.5 Hz, H-6), and δ 6.87 (d, *J =* 2.5 Hz, H-8).

**Table 1:** Summary of chromatographic retention time and mass value of TMS derivatives.

Compound	RT in GC (min)	M^+^, GC/MS TMS Derivatives	TMS Substitution Pattern
Daidzein	19.5	398	OH-group in position C7 (A-ring) and C4' (B-ring)
3',4'-*ortho*-dihydroxyisoflavone	24.1	486	OH-group in position C7 (A-ring), C3' and C4' (B-ring)
7,8-*ortho*-dihydroxyisoflavone	25.1	486	OH-group in position C7, C8 (A-ring) and C4' (B-ring)
6,7-*ortho*-dihydroxyisoflavone	26.7	486	OH-group in position C6, C7 (A-ring) and C4' (B-ring)
Glycitein	24.7	428	OH-group in position C7 (A-ring) and C4' (B-ring)

**Figure 3:**
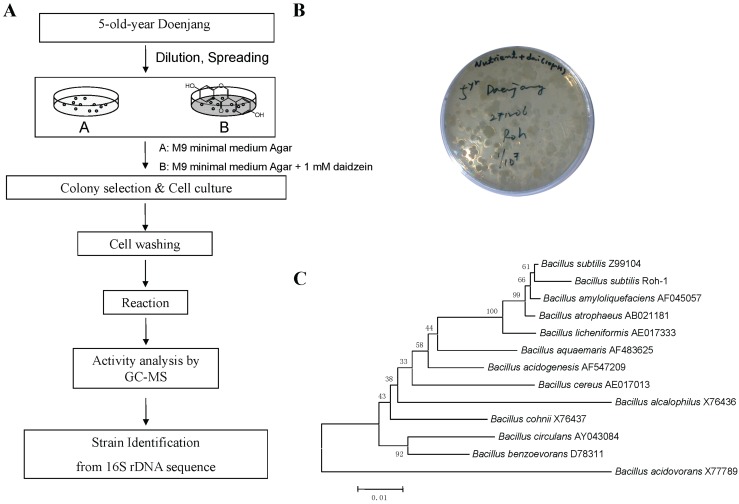
Microorganism screened from soybean Doenjang. (**A**) Strategy of strain screening from Doenjang; (**B**) Strain Roh-1 screened; (**C**) Phylogenetic tree based on 16S rRNA gene sequences showing the relationships between strain Roh-1 and other type strains. Bar, 0.01 means substitutions per nucleotide position.

**Figure 4:**
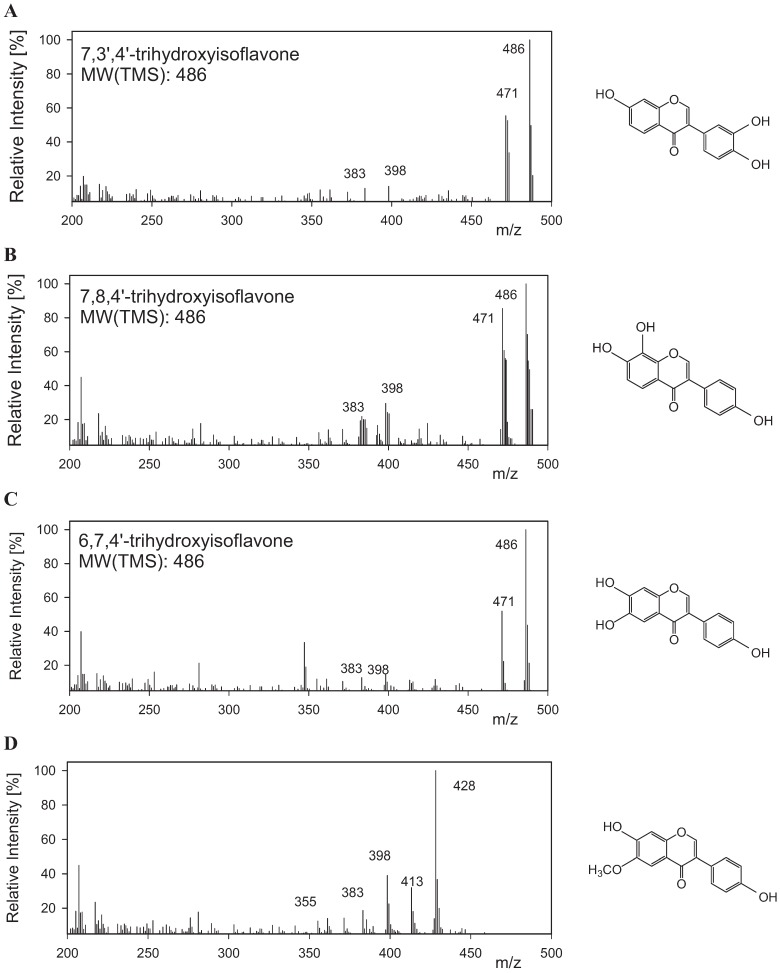
Mass spectroscopy analysis of daidzein hydroxylation from *Bacillus subtilis* Roh-1 and the chemical structures of TMS derivatives. (**A**) Mass spectra of metabolite, 7,3',4'-trihydroxyisoflavone; (**B**) Mass spectra of metabolite, 7,8,4'-trihydroxyisoflavone; (**C**) Mass spectra of metabolite, 6,7,4'-trihydroxyisoflavone; (**D**) Mass spectra of metabolite, glycitein.

### 2.3. Strain Screening Strategy from Soybean Doenjang

The strategy of the solid plate method for screening of daidzein hydroxylation using microorganisms on M9 minimal agarized media with daidzein is the capacity for hydroxylation into *ortho*-dihydroxyisoflavones. When the plates were developed without daidzein, the colonies had no daidzein hydroxylation activity. The scheme of screening for daidzein modification, producing microorganisms, and their differentiation by the characteristics of the inducible biotransformation on a solid medium plate, is shown in Figure 3. Bacterial strains showing daidzein hydroxylation activity were screened from Korean soybean Doenjang. Among bacteria screened, a strain isolated from Doenjang showed the multifunctional activity of daidzein, and was identified as *Bacillus subtilis* or *Bacillus amyloliquefaciens* by a 16S rRNA sequence analysis. The strain was designated as *Bacillus subtilis* Roh-1.

### 2.4. Biotransformation of Isoflavone Using Microorganism Screened Soybean Doenjang and Identification of Reaction Products

Among the stains screened from old soybean Doenjang, a bacterial strain showed the capability to convert daidzein into three *ortho*-dihydroxyisoflavones and glycitein in substrate specificity for regiospecific multiple hydroxylation and subsequent methoxylation with low activity. Thus, this study has for the first time demonstrated the formation of bioactive compounds using microorganisms from fermented soybean Doenjang. The analysis of the products from daidzein reaction using strain screening was confirmed using GC-MS and NMR spectroscopy, which are in good agreement (retention time, mass spectrum) with the authentic samples of *ortho*-dihydroxylated compounds and glycitein. Their compounds were also observed at *m/z* 486 for 6,7-*ortho*-dihydroxyisoflavone (RT, 26.7 min), 7,8-*ortho*-dihydroxyisoflavone (RT, 25.1 min), for 3',4'-*ortho*-dihydroxyisoflavone (RT, 24.1 min) and glycitein (7,4'-dihydroxy-6-methoxyisoflavone, RT, 24.7 min) (Figure 4).

## 3. Experimental

### 3.1. Chemicals

Daidzein, Genistein, Glycitein, 3',4'-*ortho-*dihydroxyisoflavone and 6,7-*ortho-*dihydroxyisoflavone were purchased from Sigma-Aldrich Chemical Co. (St. Louis, MO, USA). 7,8-*ortho-*dihydroxyisoflavone was obtained from Amore-Pacific Company (Yongin-si, Korea). HPLC-grade solvents were purchased form Merck (Darmstadt, Germany). *N,O*-bis(trimethylsily)trifluoroacetamide were purchased from Fluka (St. Louis, MO, USA). All other chemicals were of the highest grade available.

### 3.2. HPLC, NMR, GC-MS Analysis for Isolation

For preparative scale NMR analysis samples, the isolated sample was applied to Prep-HPLC under the following conditions: column, Alltech Econosil C18 10 U (22 × 250 mm, 5-µm particle size, Alltech Associate, Inc., Deerfield, IL, USA); UV detection, 254 nm; flow rate, 5.0 mL min^−1^; mobile phase, 100% Acetonitrile/Water (v/v) for 60 min followed by a 20%–30% Acetonitrile linear gradient, followed by 70% Acetonitrile for 40 min and followed by 80% Acetonitrile for 30 min. Samples for NMR analysis were prepared by isolating with prep-HPLC. The NMR spectra were obtained in DMSO-d6 (St. Louis, MO, USA) on a Bruker Advance 400 instrument (400 MHz, 9.4 T) (Karlsruhe, Germany). For the ^1^H-NMR experiment, 32 transients were acquired with a spectral width of 8000 Hz. All NMR data were processed using XWINNMR (Bruker, Karlsruhe, Germany). Four extracts from biotransformation of microorganism screened soybean Doenjang, *i.e.*, 6,7-*ortho-*dihydroxyisoflavone, 7,8-*ortho-*dihydroxyisoflavone, 3',4'-*ortho-*dihydroxyisoflavone, and glycitein were identified based on the interpretation of ^1^H-NMR data. For GC/MS analysis, reaction metabolites were converted to their TMS (trimethylsilyl) derivatives by incubating with BSTFA(*N,O*-bis(trimethylsily)trifluoroacetamide) for 20 min at 60 °C. GC/MS was carried out on a Finnigan MAT system (Gas chromatograph model GCQ, HP 19091J-433) connected to an ion trap mass detector. The TMS-derivatives were analyzed using a nonpolar capillary column (5% phenyl methyl siloxane capillary 30 m × 250 µm i.d., 0.25 µm film thickness, HP-5) and a linear temperature gradient (60 °C 1 min, 30 °C/min to 250 °C, held for 10 min, 1 °C/min to 275 °C, and held for 3 min). The injector port temperature was 100 °C. The scan spectrum was 100~600 m/z and the mass spectrum was obtained using electron impact ionization at 70 eV. The selected ion mode (SIM) was used for the detection of daidzein, the *ortho-*dihydroxyisoflavones, and glycitein.

### 3.3. Plate Assay Strategy

Nutrient medium (100 mL) with 1mM daidzein, at the final concentration, was prepared. The plate was solidified by the addition of agar. After diluting Doenjang (1 g) with a nutrient medium, the supernatant was spread on an agar plate with daidzein. For the control, the same experiment was conducted without daidzein. Morphologically distinct colonies were formed in Petri dishes at a density of 50 colonies per plate and the colonies were subsequently selected for liquid cultivation.

### 3.4. Cultivation and Isolation of Product from Reaction

Various kinds of strains were obtained from 5-year-old soybean Doenjang. All colonies were cultivated with nutrient and dextrose mediums. The identified strain was cultivated on nutrient medium containing 0.3% (w/v) Beef Extract, and 0.5% (w/v) Peptone. The subculture of *Bacillus subtilis* Roh-1 was carried out at 30 °C, 100× *g* for 1 day in a test tube containing 3 mL of nutrient medium. From the subculture of 1 mL, cultivation was carried out in a 250 mL conical flask containing 50 mL of the medium. For the reaction, 1 g cell (wet weight) was added in 9 mL of nutrient medium with 1 mL of daidzein solution (1 mM in DMSO: MeOH = 3%:7%, v/v). The total reaction volume was 50 mL and was shaken for 24 h at 30 °C. After 24 h, the reactant was extracted with ethylacetate (JUNSEI, Kyoto, Japan). The supernatant was evaporated in a centrifugal vacuum concentrator (BioTron, Puchon, Korea) and derivatized in BSTFA (Fluka).

## 4. Conclusions

Doenjang is a unique traditional Korean fermented soybean paste. Wide applications of such biotransformation are possible in the development of functional soybean foods. The possibility of using our strategy to identify microorganisms for biosynthesis of *ortho-*dihydroxyisoflavones and glycitein was examined. In this study, the proposed agar plate method of screening for microorganisms producing *ortho-*dihydroxyisoflavones is not only simple but also allows differentiation between microorganisms by the character of inducible biosynthesis. This scheme is necessary in the direct screening for hydroxylation of specific compounds. The metabolic reaction identified in this study involved the hydroxylation of daidzein to 6,7-*ortho-*dihydroxyisoflavone, 7,8-*ortho-*dihydroxyisoflavone, and 3',4'-*ortho-*dihydroxyisoflavone, which might be catalyzed by oxygenases. The identified strain catalyzed daidzein to glycitein (7,4'-dihydroxy-6-methoxyisoflavone), which means that the methoxylation reaction might be a possibility and that C-hydroxylation at position 6 of the A-ring was due to the subsequent *O*-methylation of 6,7-*ortho-*dihydroxyisoflavone by *O*-methyltransferase. Among all strains screened, *Bacillus subtilis* Roh-1 astonishingly converted daidzein into three *ortho*-dihydroxyisoflavones and glycitein. The GC-MS results of the products from substrates are consistent with the expected molecular weights of the hydroxylated and methoxylated form. The recent report on the antioxidant properties of naturally occurring isoflavones and their corresponding *ortho-*dihydroxyisoflavones or methoxylated isoflavones enhances their antioxidant effects as well as biological properties [14,15,16,17,18]. To the best of our knowledge, this is first report that a bacterial strain screened from soybean Doenjang had substrate specificity for regio-specific multiple hydroxylation and methoxylation. In addition, it can be applied in aspect of biotransformation for the production of bioactive compounds. 

## References

[1] Lee S.S. (1995). Meju fermentation for a raw material of korean traditional soy products. Korea J. Mycol..

[2] Kim D.H., Kim S.H., Choi N.S., Bai S., Chun S.B. (1998). Biochemical characteristics of whole soybean cereals fermented with *Aspergillus strains*. Korea J. Appl. Microbiol. Biotechnol..

[3] Chung D.H., Shim S.K. (1994). Soybean Fermentation Food.

[4] Kwon T.W., Song Y.S., Kim J.S., Moon G.S., Kim J.I., Hong J.H. (1998). Current research on the bioactive functions of soyfoods in Korea. Korea Soybean Digest..

[5] Pratt D.E., Birac P.M. (1979). Sources of antioxidant activity of soybeans and soy products. J. Food Sci..

[6] Coward L., Barnes N.C., Setchell K.D.R., Barnes S. (1993). Genistein, daidzein, and their beta-glycoside conjugates: Antitumor isoflavones in soybean foods form American and Asian diets. J. Agric. Food Chem..

[7] Wang H., Murphy P. (1994). Isoflavone content in commercial soybean foods. J. Agri. Food Chem..

[8] Eldridge A.C., Kwolek W.F. (1983). Soybean isoflavones: Effect of environment and variety on composition. J. Agri. Food Chem..

[9] Dixon R.A. (2004). Phytoestrogens. Annu. Rev. Plant Biol..

[10] Choi J.S., Kwon T.W., Kim J.S. (1996). Isoflavone contents in some varieties of soybean. Foods Biotech..

[11] Foti P., Erba D., Riso P., Spadafranca A., Criscuoli F., Testolin G. (2005). Comparison between daidzein and genistein antioxidant activity in primary and cancer lymphocytes. Arch. Biochem. Biophy..

[12] Kim H.W., Ko E.J., Ha S.D., Song K.B., Park S.K., Chung D.H., Youn K.S., Bae D.H. (2005). Physical, mechanical, and antimicrobial properties of edible film produced from defatted soybean meal fermented by *Bacillus subtilis*. J. Microbiol. Biotechnol..

[13] Lim J.S., Jang C.H., Lee I.A., Kim H.J., Lee C.H., Kim J.H., Park C.S., Kwon D.Y., Lim J., Hwang Y.H. (2009). Biotransformation of free isoflavones by *Bacillus* species isolated from traditional *cheonggukjang*. Food Sci. Biotech..

[14] 1Klus K., Barz W. (1995). Formation of polyhydroxylated isoflavones from the soybean seed isoflavones daidzein and glycitein by bacteria isolated from tempe. Arch. Microbiol..

[15] Kulling S.E., Honig D.M., Metzler M. (2001). Oxidative metabolism of the soy isoflavones daidzein and genistein in humans *in vitro* and *in vivo*. J. Agric. Food Chem..

[16] Rufer C.E., Kulling S.E. (2006). Antioxidant activity of isoflavones and their major metabolites using different *in vitro* assays. J. Agric. Food Chem..

[17] Fujita T., Funako T., Hayashi H. (2004). 8-hydroxydaidzein, an aldose reductase inhibitor from Okara fermented with *Aspergillus* sp. HK-388. Biosci. Biotechnol. Biochem..

[18] Chen Y.C., Sugiyama Y., Abe N., Kuruto-Niwa R., Nozawa R., Hirota A. (2005). DPPH radical-scavenging compounds from dou-chi, a soybean fermented food. Biosci. Biotechnol. Biochem..

